# Novel Oliveros-like Clade C *Mammarenaviruses* from Rodents in Argentina, 1990–2020

**DOI:** 10.3390/v16030340

**Published:** 2024-02-22

**Authors:** Elizabeth Shedroff, Maria Laura Martin, Shannon L. M. Whitmer, Julia Brignone, Jorge B. Garcia, Carina Sen, Yael Nazar, Cintia Fabbri, Maria Morales-Betoulle, Jairo Mendez, Joel Montgomery, Maria Alejandra Morales, John D. Klena

**Affiliations:** 1 Viral Special Pathogens Branch, The Centers for Disease Control and Prevention, 1600 Clifton Rd., Atlanta, GA 30329, USA; ubu2@cdc.gov (E.S.); evk3@cdc.gov (S.L.M.W.); fof7@cdc.gov (M.M.-B.); ztq9@cdc.gov (J.M.); 2 Instituto Nacional de Enfermedades Virales Humanas Dr. Julio I. Maiztegui, Monteagudo 2510, Pergamino 2700, Argentina; malalamar@gmail.com (M.L.M.); jubrignone@yahoo.com.ar (J.B.); jorgebgarcia@gmail.com (J.B.G.); carinanoesen@gmail.com (C.S.); ynazar@anlis.gob.ar (Y.N.); cintiafabbri@yahoo.com.ar (C.F.); morales.mariaalejandra@yahoo.com.ar (M.A.M.); 3 Pan American Health Organization, 525 23rd St. New World, Washington, DC 20037, USA; ricoj@paho.org

**Keywords:** *Mammarenaviruses*, Argentina, *Necromys*, phylogenetics, sequencing

## Abstract

Following an Argentine Hemorrhagic Fever (AHF) outbreak in the early 1990s, a rodent survey for Junín virus, a New World Clade B arenavirus, in endemic areas of Argentina was conducted. Since 1990, INEVH has been developing eco-epidemiological surveillance of rodents, inside and outside the Argentine Hemorrhagic Fever endemic area. Samples from rodents captured between 1993 and 2019 that were positive for Arenavirus infection underwent Sanger and unbiased, Illumina-based high-throughput sequencing, which yielded 5 complete and 88 partial *Mammarenaviruses* genomes. Previously, 11 genomes representing four species of New World arenavirus Clade C existed in public records. This work has generated 13 novel genomes, expanding the New World arenavirus Clade C to 24 total genomes. Additionally, two genomes exhibit sufficient genetic diversity to be considered a new species, as per ICTV guidelines (proposed name *Mammarenavirus vellosense*). The 13 novel genomes exhibited reassortment between the small and large segments in New World *Mammarenaviruses*. This work demonstrates that Clade C *Mammarenavirus* infections circulate broadly among *Necromys* species in the Argentine Hemorrhagic Fever endemic area; however, the risk for Clade C *Mammarenavirus* human infection is currently unknown.

## 1. Introduction

*Mammarenaviruses* are rodent-borne ambisense RNA viruses that contain large (L) and small (S) genome segments. The S segment encodes for the nucleoprotein (NP) and glycoprotein (GPC), whereas the L segment encodes for the polymerase (L) and zinc finger (Z) proteins [1]. Virions are surrounded by a lipid bilayer containing glycoprotein spikes required for cellular entry and internally contain viral segments coated in nucleocapsid, the viral polymerase required for replication, and the Z protein for virion budding [2]. The Mammarenavirus genus is split into two major groups: Old World (OW) lymphocytic choriomeningitis (LCM) serocomplex and New World (NW) Tacaribe serocomplex [3]. New World *Mammarenaviruses* are divided into four lineages: Clade A, Clade B, Clade C, and a tentative Clade D, sometimes also called Clade A–B [4]. Currently, Clade C contains only three viral species and has the smallest lineage of all New World clades [5]. Arenaviruses are relatively understudied due to the isolation of their host species to rural areas [6]. 

Nearly all *Mammarenaviruses* have rodent hosts, except for Tacaribe virus, which is carried by *Artibeus lituratus palmorum* and *Artibeus jamaicensis* bat species [7,8]. The evolution of arenaviruses is driven by three major mechanisms: point mutations, reassortment, or recombination [2]. Historically, there has been limited supporting evidence of reassortment in nature, but reassortment has been demonstrated in vitro [9,10]. Reassortment is achieved by co-infection in either a natural reservoir or cell culture [2]. Although it is uncommon, there are documented cases of Arenavirus co-infection among sympatric rodent hosts [11]. South American New World *Mammarenaviruses* that cause human disease have been identified in *Akodon*, *Calomys*, *Necromys*, *Oryzomys*, Oligoryzomys, and *Sigmodon* rodent species [12]. *Mammarenavirus* species are hypothesized to have co-evolved with their rodent hosts, resulting in the association of a single *Mammarenavirus* species with a single rodent species [13,14]; however, more recent data suggest that cospeciation is unlikely in *Mammarenaviruses* and that the associations between *Mammarenavirus* species and their reservoirs is more nuanced than previously thought [14,15]. 

*Mammarenaviruses* typically cause a chronic, asymptomatic infection in their primary hosts [7]. Several Clade B viruses and one Clade A virus are transmissible to humans [16,17]. In humans, Arenavirus infections are symptomatic and manifest as viral hemorrhagic fevers (VHFs) [16]. Junín virus is the causative agent for Argentine Hemorrhagic Fever (AHF) and is endemic to the rural regions in central Argentina [18,19]. South American New World *Mammarenaviruses* are also responsible for Bolivian hemorrhagic fever (Machupo virus), Chapare hemorrhagic fever in Bolivia (Chapare virus), Venezuelan hemorrhagic fever (Guanarito virus), hemorrhagic fever in Brazilian laboratory workers (Flexal virus), asymptomatic infection in Colombian researchers (Pichinde virus), Southwestern US hemorrhagic fevers (Whitewater Arroyo virus), and Brazilian hemorrhagic fever (Sabia virus) in addition to AHF [13,17,20,21,22]. Infections can present with flu-like symptoms, making diagnosis difficult if there are no known outbreaks at the time of symptoms [23]. Early symptoms typically present as fever, malaise, headache, myalgia, and gastrointestinal distress, then progress to neurological involvement such as meningitis and encephalomyelitis [24,25]. Without treatment, the case fatality rate (CFR) of South American hemorrhagic fevers such as Argentine Hemorrhagic Fever, Bolivian Hemorrhagic Fever, and Venezuelan Hemorrhagic fever, can reach 30% [25]. Moreover, because these nonspecific symptoms can mimic endemic diseases like dengue, there is typically limited case detection in remote regions.

Following an AHF outbreak in the 1980s, rodents in Argentina were surveyed to investigate the dynamics of Junín virus [26,27]. During this survey, Oliveros virus was identified as a distinct species within Clade C. Despite the regional proximity, phylogenetic analysis revealed a distant relationship between Junín and Oliveros viruses [28]. Oliveros virus, like many other *Mammarenaviruses*, are also hypothesized to have co-evolved and restrict themselves to specific hosts and regions due to the isolated nature of their local habitats [6,29]. Pampa virus was also identified during this time period, a novel New World Clade C Arenavirus for which only a partial S segment exists [30]. 

Since 1990, the Argentinian Instituto Nacional de Enfermedades Virales Humanas (INEVH) has been conducting eco-epidemiological surveillance of *Calomys musculinus* and other rodents, inside and outside the AHF endemic area. Additionally, since the emergence of Hantavirus Pulmonary Syndrome (HPS), INEVH has assisted in more than 50 outbreaks of HPS by sampling rodents in peridomestic and countryside areas in four regions of Argentina. This extensive animal surveillance effort allows for the monitoring of viruses that circulate in rodent communities not originally under investigation, improving surveillance and pathogen discovery in the absence of an active outbreak.

Here, additional Clade C *Mammarenavirus* sequences were generated, consisting of one partial large (L) segment, seven partial small (S) segments, and five full-length L and S segments. Phylogenetic analysis revealed that these *Mammarenavirus* genomes expand the New World *Mammarenavirus* Clade C from 11 genomes to 24. Additionally, two genomes exhibit sufficient genetic diversity to be considered new species, as per the ICTV guidelines (proposed name, Vello virus, species *Mammarenavirus vellosense*). The 13 novel genomes exhibited reassortment between the S and L segments in New World *Mammarenaviruses*. 

## 2. Materials and Methods

### 2.1. Rodent Collection and RNA Extraction

Rodent field sampling occurred during HPS outbreaks or annually in mid-autumn to take advantage of population abundance and historically better trap success rates. Live traps were placed in each site for three days using multiple-trap lines; a minimum of 300 traps per night were set. Captured rodents were identified by species, sex, and age.

Blood samples were taken by cardiac puncture or retro-orbital sinus from anesthetized animals, then terminal tissue samples (lung, liver, spleen) were taken after euthanasia. Blood and tissues were immediately placed in liquid nitrogen for transport to long-term storage for virological testing. 

Rodent RNA samples were obtained from INEVH and extracted according to Chomcznsky et al. [31]. At the Viral Special Pathogens Branch laboratory (CDC, Atlanta, GA, USA), RNA was extracted using TriPure (Roche, San Francisco, CA, USA) with phase separation using 1-bromo-3-chloropropane. The upper phase was applied to RNeasy-25 columns to extract the RNA (Zymo, Irvine, CA, USA). 

### 2.2. Identification of Arenavirus-Positive Samples

Rodent samples were tested via serological methods or conventional PCR to determine the presence of New World arenaviruses. Pan-arenavirus primers were utilized with a conventional RT-PCR assay to identify arenavirus-positive rodents [28,30]. Briefly, RT-PCR screening was accomplished with 19C, a generic New World *Mammarenavirus* S segment primer, and 16V, a primer to amplify a 600 nucleotide (nt) fragment of the nucleoprotein genomes New World [32]. Screening was accomplished via one-step RT-PCR; therefore, the 1010C and 1696R primers were not utilized per Bowen et al. [33]. Amplicons from these RT-PCR reactions were sequenced to generate partial viral genomes.

For IgG antibody detection, an enzyme-linked immunoassay (ELISA) test was carried out using specific antigens for JUNV and Latino virus (LATV) following previously published protocols [33,34]. 

### 2.3. High-Throughput Sequencing and Bioinformatics

Next-generation sequencing libraries were generated by treating RNA with RNase-free DNase-I (Roche, San Francisco, CA, USA). Ten microliters of RNA was diluted with 35 μL nuclease-free water, mixed with 5 uL 10× DNAse 1 RNAse-free incubation buffer and 1 uL DNAse, and incubated at room temperature for 15 min, followed by a 2.2× RNA XP SPRI bead cleanup. The library was prepared using an NEBNext Ultra II Directional RNA Library Preparation kit (NEB, Ipswich, MA, USA). The library was sequenced using a MiSeq with a 300-cycle cartridge (Illumina, San Diego, CA, USA). 

Guided de novo assembly was performed at CDC and INEVH and contigs were identified using in-house scripts. At INEVH, raw data were processed with fastp (v0.23.4), de novo assembled with SPAdes (v3.15.5), and duplicates were removed with Fastunique (v1.1) [35,36,37]. At CDC, reads were trimmed for quality with prinseq-lite (-min_qual_mean 25-trim_qual_right 20-min_len 50), and contigs were assembled using SPAdes (-k auto) (v3.14.0). Contigs were blasted to the non-redundant Genbank database using in-house Python scripts running Python (v3.8.5) to identify the most closely related reference sequence (https://github.com/evk3/hantavirus_US_distribution#bioinformatics accessed on 11 August 2023). The first Clade C *Mammarenavirus* (Vello virus) was assembled by iteratively mapping to the Oliveros reference sequence (NC_010248). Subsequent arenavirus genomes were generated by mapping contigs and trimmed reads to full-length Clade C arenaviruses (Oliveros, Vello, Pampa, and Ura viruses) using the Geneious mapper (high sensitivity/slow) (Geneious Prime 2022.0.2). Sequences generated at CDC and INEVH exhibited greater than 96% nucleotide identity, and differences/gaps were resolved by selecting the SNP with the highest coverage (Tables S1 and S2).

Nucleotide and amino acids were aligned using MAFFT v7.490 (--auto --thread $NSLOTS) in Geneious Prime 2022.0.2. Percent nucleotide identity and amino acid similarity (Blosum62 cost matrix) for S and L segments were calculated using the Geneious Prime 2022.0.2. Nucleotide-based trees were constructed using RAxML v8.2.12-PTHREAD (-m GTRGAMMAI-p $RANDOM-f a-x $RANDOM-T $NSLOTS-PTHREADS-N 1000). Amino-acid based trees were generated using RAxML v8.2.12 -PTHREAD (-m PROTGAMMAIAUTO-p $RANDOM-f a-x $RANDOM-T $NSLOTS-N 1000). Phylogenetic trees were visualized using ggtree, ggplot2, RColorBrewer, and phangorn (R v4.1.3) with JupyterHub v1.9.1. The tanglegram was generated by comparing phylogenetic trees generated using full-length sequences for Clade C L segments and S segments and tanglegram connections added by hand. Full-length and partial genomes are accessible on Genbank, Accesion numbers OR844393–OR844410.

### 2.4. PCR and Amplicon-Based Sequencing

Vello-specific Z-protein primers 6918F (AAT CAT CAC CCC GGC ATA GC), 7359R (CGG GGA TCC TAG GCA CAA G), L-protein primers 5434F (CAA CTT GAG GAG AGG CTG GG), 6076R (CAG CAA AGT CAC ACG GAA GC), and whole S-segment primers 2861F (CAA GCG TGG TCA AAC CTT GG), 3420R (GTC ACT GAG GCG AGG TTT GA) were designed in Geneious Prime following Vello virus genome assembly. RT-PCR was performed using a Biorad PCR machine and the LunaScript RT SuperMix Kit (NEB, Ipswich, MA, USA) with 10 µM forward and 10 µM reverse primers. Cycle conditions consisted of 10 min at 55 °C for reverse transcription, 60 s at 98 °C for RT inactivation and 40 cycles of 10 s at 98 °C for denaturation, 10 s at 68 °C for annealing, 25 s at 72 °C for extension, with 5 min at 72 °C for final extension. PCR products for the Z-protein (441 bp), L-protein (642 bp) and S segment (559 bp) were separated on a 2% E-Gel to confirm the presence of Vello virus-specific amplicons (ThermoFisher, Waltham, MA, USA). Vello virus amplicons were sequenced using the Ligation Sequencing Kit (v12) and the Mk1B MinION instrument (ONT, Oxford, UK). Basecalling and demultiplexing was performed using guppy (v6.4.6).

## 3. Results

### 3.1. Identification of Novel Clade C Mammarenaviruses from Field Collected Rodents 

**Figure 1:**
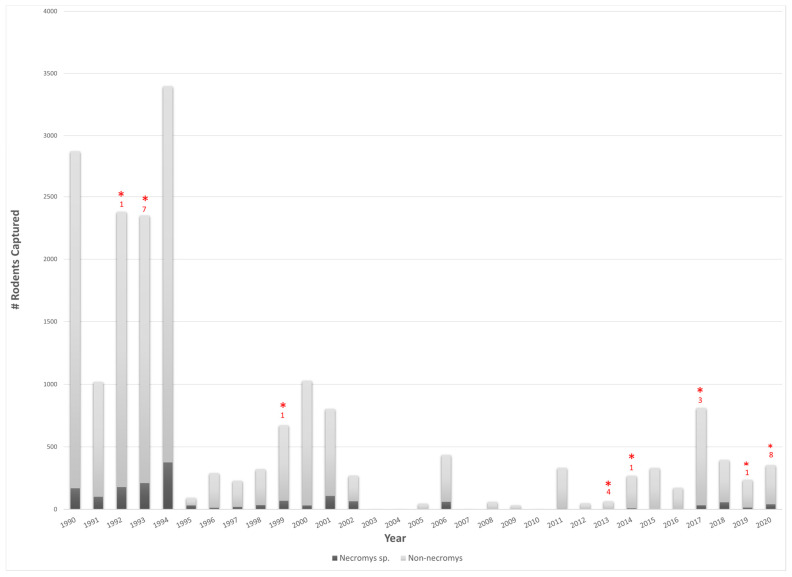
Rodent surveillance from 1990 from 2020 in Argentine Hemmohagic Fever endemic regions of Argentina. Bars represent the number of rodents captured. Dark gray represents *Necromys* species captured, and light gray represents total number of non-Necromys rodents captured. Red asterisks (*) indicate a year in which arenavirus-positive samples were identified. The number of *Mammarenavirus*-positive rodents captured per year is shown in red on the top of each bar. Overall, 1890 rodents were captured between 1990 and 2020, and of those rodents, 26 were identified as positive, yielding a 1.38% *Mammarenavirus* positivity case rate.

Following rodent collection in endemic areas of Argentina, humane euthanasia, and autopsy, total RNA was extracted from rodent brain and/or lung tissue and screened for the presence of arenaviruses using pan-New World arenavirus conventional RT-PCR (Figure 1 and Figure S1, Table S3). Alternatively, some rodents (*n* = 14) were screened for the presence of anti-arenavirus antibodies by ELISA (Table S3). Overall, 1890 *Necromys* species were captured between 1990 and 2020, and of those rodents, 26 were identified as positive by either method, resulting in a 1.65% positive case rate (Figure 1 and Supplemental Table S1). Samples identified as positive by RT-PCR and/or ELISA (*n* = 26) were sequenced using Sanger (*n* = 9) and/or high-throughput sequencing (*n* = 26) which resulted in five complete genomes, 77 partial S segments, and one partial L segment from 13 different samples (Supplemental Table S1). Overall, 50% of the identified positive samples yielded sequence data. 

The complete genome for Vello virus (isolate 13796) was the first novel *Mammarenavirus* that was sequenced in this work and was identified in a Maciel hantavirus isolate, originally generated following the passage of *Necromys obscurus* lung tissue in Vero E6 cells. The presence of Vello virus was confirmed by RT-PCR and sequencing; in vitro sequencing results were 99–100% identical to the Vello virus genome generated in silico (Figure S2, Table S4). The Vello virus genome (and Oliveros and Pampa genomes) were used as reference sequences when building genomes from the 26 Arenavirus-positive rodents.

**Figure 2:**
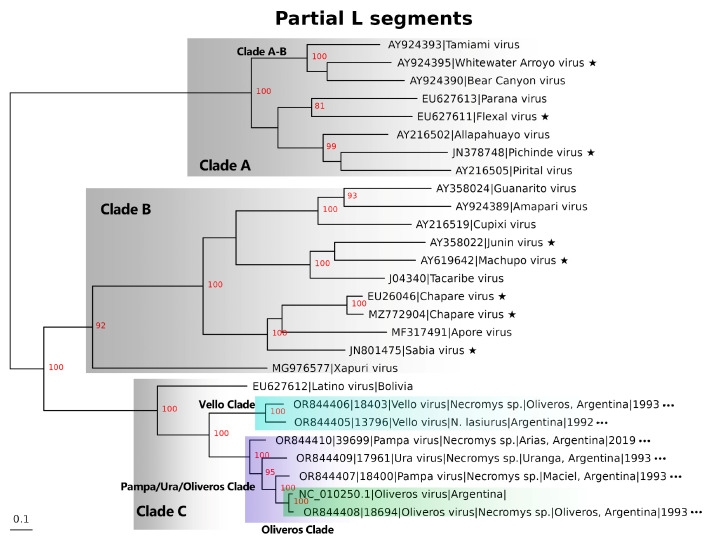
Inferred phylogenetic relationships generated by maximum likelihood using partial *Mammarenavirus* L nucleotide segments (1744/7472 bp). Major clades are labeled (A–B through C), and minor clades in Clade C are highlighted, trees are midpoint rooted, and bootstrap support (*n* = 1000 iterations) is highlighted in red on each node. Black stars indicate New World *Mammarenaviruses* known to cause human infections. Three dots indicate sequences generated in this work. Purple highlighting indicates the Pampa/Ura/Oliveros virus clade, green highlighting outlines the Oliveros virus clade, and turquoise highlighting outlines the Vello virus clade.

**Figure 3:**
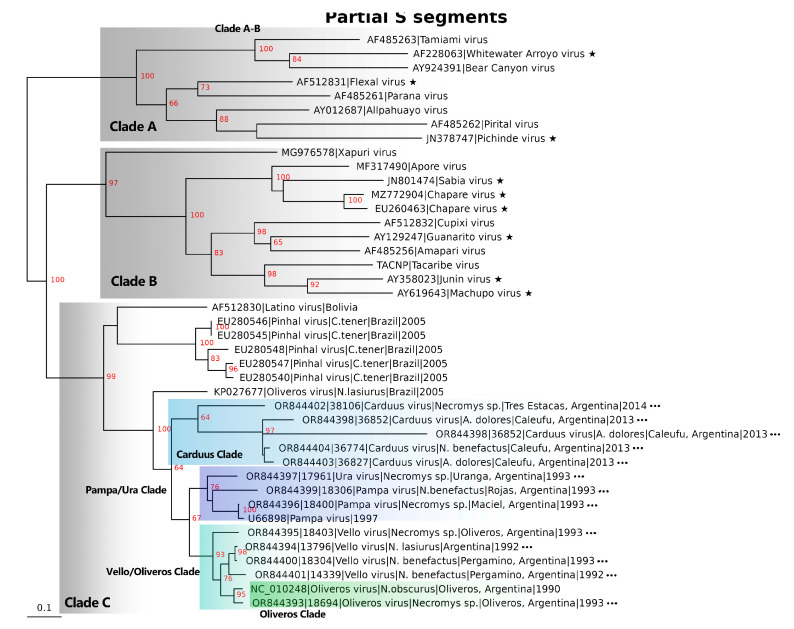
Inferred phylogenetic relationships generated by maximum likelihood using partial New World *Mammarenavirus* S nucleotide segments (1089/4184 bp). Major clades are labeled (A–B through C), and minor clades in Clade C are indicated (highlights), trees are midpoint rooted, and bootstrap support (*n* = 1000 iterations) is highlighted in red on each node. Black stars indicate New World *Mammarenaviruses* known to cause human infections. Three dots indicate sequences generated in this work. Light blue highlighting represents that Carduus virus clade, purple highlighting indicates the Pampa/Ura virus clade, turquoise highlighting indicates the Vello/Oliveros virus clade, and green highlighting outlines the Oliveros virus clade.

Using complete and partial sequences, phylogenetic relationships were inferred between new and existing *Mammarenaviruses* (Figure 2, Figure 3 and Figures S3–S5). The sequences generated in this work clustered within Clade C with strong bootstrap support. The nucleotide-based L segment tree displays 100% bootstrap support for Vello virus’ ancestral relationship to a Ura/Pampa/Oliveros Clade (Figure 2, purple highlight). Amino acid-based trees also show a well-supported outgrouping of the Vello virus clade relative to the Ura/Pampa/Oliveros Clade (Figure S5A). The S segment nucleotide-based tree demonstrates 100% bootstrap support for an ancestral relationship between Oliveros virus in Brazil (KP027677) and the Argentinian New World Arenaviruses. In contrast to the L segment trees, the Argentinian S segment is subdivided into a Carduus Clade (blue highlight), Pampa/Ura Clade (purple highlight), and Vello/Oliveros Clade (turquoise highlight) (Figure 3). Amino acid-based trees agree with the nucleotide-based trees and also show a well-supported separation between the Pampa/Ura Clade (purple highlight), Vello/Clade (turquoise highlight) (Figure S5B).

### 3.2. Evidence of Reassortment/Diversity

**Figure 4:**
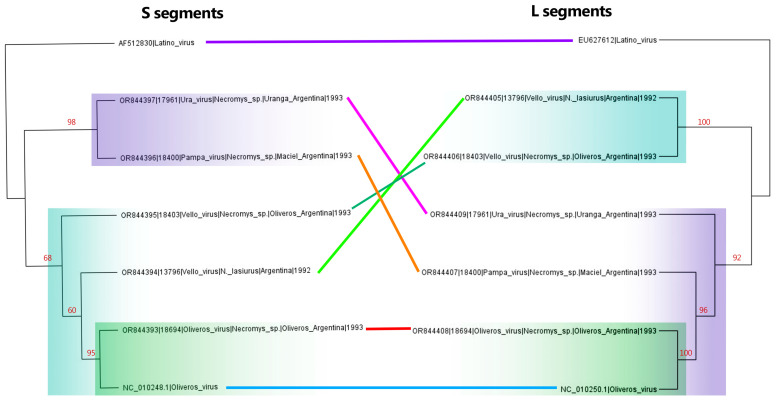
Tanglegram of complete *Oliveros*, *Vello*, and *Latino* species S and L nucleotide segments. Lines connecting phylogenetics trees are colored by each sample. Trees are midpoint-rooted. Bootstrap support (*n* = 1000 iterations) is highlighted in red on internal nodes. Purple highlighting indicates the Pampa/Ura virus Clade, turquoise highlighting indicates the Vello virus-containing clade, and green highlighting outlines the Oliveros virus clade.

To better clarify the inferred relatedness of Clade C *Mammarenaviruses*, tanglegrams comparing the full-length S and L segments were constructed (Figure 4). In the S segments, Vello virus (turquoise highlight) appears to be ancestral to Oliveros virus (green highlight) while the Pampa and Ura viruses (purple highlight) are closely related and form an outgroup. In contrast, the L segments for Pampa and Ura virus (purple highlight) are ancestral to Oliveros virus (green highlight), and Vello virus (turquoise highlight) forms an outgroup. Evidence of reassortment was observed between the S and L segments of Vello virus (isolates 18403 and 13796), Ura virus (isolate 17961), and Pampa virus (isolate 18400) (Figure 4).

### 3.3. Identification of Novel Species Vello Virus and Other Inter-Species Diversity

The International Committee for Taxonomy of Viruses (ICTV) delineates that a new *Mammarenavirus* species should exhibit less than 88% NP amino acid identity, less than 80% S segment nucleotide identity and less than 76% L segment nucleotide identity compared to existing viruses [38]. In instances where only one segment meets the threshold criteria or when viral relatedness is uncertain, ICTV’s Arenavirus Study Group (ASG) recommends using the diversity of the L segment for delineating novel virus species [39]. 

Based upon the L segment nucleotide percent identity, Vello virus isolates 18403 and 13796 appear to belong to the same species and meet species demarcation criteria among other *Mammarenaviruses* (Table 1 and Table S5). These specimens are assigned the proposed species name of “*Mammarenavirus vellosense*.” In contrast, the Pampa, Oliveros, and Ura virus L segments exhibit greater than 83% similarity and are members of *Mammarenavirus oliverosense*. The S segments for Ura, Vello, Pampa, and Oliveros viruses are greater than 82% identical. However, while the Ura virus S segment (isolate 17961) has a nucleotide percent identity of 72–75% with related viruses, suggesting that it could represent a novel species, it is 83% identical to Oliveros virus (isolate 18694) and does not meet the threshold for NP amino acid similarity, indicating it is a member of *Mammarenavirus oliverosense* rather than a member of a new species (Table 1 and Table S6). 

## 4. Discussion

This work identifies complete and partial genome sequences that expand the New World *Mammarenaviruses* Clade C. Previously, one full-length Oliveros virus reference genome, one full-length Latino virus reference genome, and nine partial S segments (Oliveros, Pampa, and Pinhal viruses) existed in public records [40]. Deep sequencing of rodent samples from Argentina aimed to build a more comprehensive understanding of Clade C *Mammarenaviruses*, with the addition of five complete genomes and 8 partial genomes. The expansion of Clade C resulted in the identification of a new species and revealed evidence of reassortment in *Mammarenaviruses*.

Historically, *Mammarenavirus* species were associated with one rodent host species in a specific geographic region, and there was limited evidence of viral reassortment. Previously, Arenavirus co-infection and reassortment had only been described in snake Arenaviridae (*Reptarenaviruses*) [41], but more recently, Cuypers, et al. described an instance of Old World *Mammarenavirus* co-infection where one S segment and two diverse L segments were detected in a rodent (*Lemniscomys rosalia*) specimen in Tanzania [42]. The co-infection observed in Tanzanian rodents is the first such instance in Old World *Mammarenaviruses* but is yet to be described in New World *Mammarenaviruses* [41,42]. Here, a diverse set of New World *Mammarenaviruses* was observed to infect similar rodent species (*A. dolores*, *N. benefactus* and *C. tener*). Among these rodent species, multiple Clade C *Mammarenaviruses* can be found in the same host species, suggesting that the opportunity for co-infection and potential viral reassortment exists. Mills et al. and Fernandes et al. described a similar trend where Oliveros virus was found in multiple *Necromys* species [5,43]. Opportunities for co-infection are supported by the shared geographic location of diverse *Mammarenaviruses*, such as Junin and Oliveros virus in Argentina [44].

Additionally, Araraquara, Juquitiba and Maciel hantaviruses also circulate among *Necromys* and *Akodon* rodents [45,46]. Choclo hantavirus, Lechiguanas hantavirus and Chapare arenavirus also share an *Oligoryzomys* host [47,48]. Furthermore, one species of hantavirus can infect multiple rodent hosts, which is similar to the observed *Mammarenavirus* dynamics in this study [45]. Co-infections between Arenaviridae and Hantaviridae are rare, but can occur naturally within shared rodent host species, as shown by the seroprevalence of Maciel virus and Junin virus in Colombia [49]. There have also been documented co-infections between Old World *Mammarenaviruses* and *Orthohantaviruses*, as shown by the presence of both lymphocytic choriomeningitis virus (LCMV) antibodies and *Orthohantavirus* antibodies in rodents from Italy and Spain [50,51]. 

In addition to rodents, humans are also at risk for Hantavirus and Arenavirus co-infection, as there have been two documented cases in which antibodies against Sin Nombre virus and either Guanarito or Whitewater Arroyo virus were detected in rodent-exposed North American workers [52]. Hantavirus and Arenavirus human infection is typically facilitated by occupational contact with rodents, which includes inhalation of infectious aerosols or animal bites [53]. Interestingly, *Calomys musculinus*, the rodent host for Junín virus, does not exhibit behavior that would be conducive to occupational exposure. The preferred habitats for these rodents are fence lines or roadside field borders, rather than the fields themselves [53]. Due to similar rodent hosts with complex dynamics, significant disease pathologies, and shared geographic regions, there may be utility in conducting paired surveillance for *Mammarenaviruses* and *Orthohantaviruses.*

New World *Mammarenaviruses* within Clades A and B are known to cause human disease, but it is currently unknown as to whether Clade C viruses can cause disease too. The recent Chapare virus outbreak in Bolivia highlights that there are still challenges to investigating Arenavirus cases, even when the virus is already known to cause human infections [20,21]. Most arenavirus endemic areas tend to be in isolated, rural regions with limited resources [54]. Non-specific clinical criteria may also contribute to potential under-reporting of arenaviral hemorrhagic fevers [13]. Improved rodent surveillance for arenaviruses within and around these geographic regions could improve diagnostics and risk evaluations for arenaviral hemorrhagic fevers. 

Clade C *Mammarenaviruses* are unique from Clades A, B and A–B in that they share a major receptor (α-Dystroglycan, or α-DG) and coreceptor with Guanarito virus (etiological agent for Venezuelan hemorrhagic fever) and Old World *Mammarenaviruses* already associated with Lassa fever and Lymphocytic choriomeningitis [13,55]. Pathogenic Clade *B Mammarenaviruses* use transferrin receptor 1 (TfR1) as their major receptor, which may indicate that Clade C *Mammarenaviruses* retained the α-DG phenotype while other New World *Mammarenaviruses* evolved to use a different entry method [56,57]. 

In addition to receptor usage, intraclade reassortment may also increase the risk of future hemorrhagic fever disease, especially given that Clade A, B, and C viruses co-circulate in the same geographic regions. Notably, co-infection between Clade A and Clade B arenaviruses has been documented in Venezuela [11]. Despite well-documented instances of co-infection, recombination between New World *Mammarenaviruses* has rarely been described in nature, which may be explained by superinfection exclusion [58,59,60]. Xapuri virus may represent an occurrence of natural reassortment between New World *Mammarenaviruses* due to its phylogenetic relationship to Clade B and Clade C [61].

More deep sequencing of historic *Mammarenavirus*-positive samples would be useful to expand our knowledge of arenaviruses. Improved viral surveillance would aid the identification new species of arenaviruses before VHF outbreaks occur. Clade C, particularly, is the smallest and most understudied clade of *Mammarenavirus* and by expanding Clade C, this work demonstrates the possibility that additional species exist. For example, the addition of novel sequences demonstrates that the Brazilian and Argentinian Oliveros viruses are more distantly related than previously thought. Moreover, viral species are often characterized not only by their geographic distribution, but also the disease they are associated with [38]. The viral L segment not only serves as a basis for species characterization, but is also a determinant of virulence in rodent models [62]. Currently, ICTV guidelines describe species demarcation criteria that rely on both the L and S segments, but the data shown here demonstrate that only one out of the two segments can meet these criteria, which could represent an intermediate stage of speciation. Future re-assessment of the taxonomic criteria may be necessary to accommodate new information generated by sequencing. Additionally, speciation, natural selection, and genetic drift of *Mammarenaviruses* may be clearer with more sequence data over this timespan. For example, Vello virus and Oliveros virus were predominantly detected between 1992 and 1993, while Carduus virus was detected between 2013 and 2014, but it is unclear if this is evidence for selection, or due to rodent convenience sampling.

There are limitations to the phylogenetic analyses presented in this work, as they are based upon some partial sequences, for example, Carduus virus. Future assessments of Clade C *Mammarenaviruses* may involve viral culturing of the isolates presented here to yield full-length genomes. Additionally, the rodent surveillance in this work took place in northern regions of Argentina, which may not necessarily represent the full *Mammarenavirus* distribution in the country. Future studies may benefit from extending surveillance beyond Argentine Hemorrhagic Fever endemic areas to capture more diverse *Mammarenavirus* species and better understand their geographic distribution. The findings presented here can be used to direct additional studies in Argentina to address these limitations.

## 5. Conclusions

A twenty-year-long *Mammarenavirus* surveillance effort in rodents from hemorrhagic fever endemic regions of Argentina and subsequent sequencing yielded five complete and eight partial *Mammarenaviruses* genomes that expand Clade C. The complete genomes exhibited reassortment between the viral S and L segments, which highlights important observations in rodent dynamics, geographic distribution, and implications for human disease. Additionally, two *Mammarenavirus* genomes in this work exhibit enough L segment diversity to define a new species, with the proposed name “Vello virus”, species “*Mammarenavirus velloense*”. Clade C is a historically understudied group of *Mammarenaviruses* that has distinct and unique characteristics unlike other New World *Mammarenaviruses.* Improved surveillance and sequencing efforts may produce the data necessary to determine if Clade C *Mammarenaviruses* can cause human disease, demonstrate potential genetic drift and more instances of reassortment, or further identify what makes them unique from Clades A, B and A–B.

## Figures and Tables

**Table 1:** Full-length Arenavirus species demarcation. Viruses marked with a red diamond indicate proposed name for a new species. Checkmarks and green highlighting indicate that a genome has satisfied species demarcation criteria. Asterisk indicates that the sequence meets species demarcation against all but one other sequence.

	Oliveros Virus, 18694 S: OR844393 L: OR844408	Vello Virus✦, 13796 S: OR844394 L: OR844405	Vello Virus✦, 18403 S: OR844395 L: OR844406	Pampa Virus, 18400 S: OR844396 L: OR844407	Ura Virus✦, 17961 S: OR844397 L: OR844409
Meets S segment criteria <80% nucleotide identity	✖	✖	✖	✖	✔ *
Meets NP criteria <88% amino acid similarity	✖	✖	✖	✖	✖
Meets L segment criteria <76% nucleotide identity	✖	✔ (same species as 18403)	✔ (same species as 13796)	✖	✖

## Data Availability

Genome sequences can be found on NCBI’s GenBank.

## Supplementary Materials

The following supporting information can be downloaded at: https://www.mdpi.com/article/10.3390/v16030340/s1, Figure S1: Rodent distribution map; Figure S2: Vello virus gel; Figure S3: Full L segment tree; Figure S4: Full S segment tree; Figure S5: Amino acid-based protein trees; Table S1: L segment nucleotide percent similarity between INEVH and CDC sequences; Table S2: S segment nucleotide percent similarity between INEVH and CDC sequences; Table S3: Sample metadata; Table S4: Vello sequence in vitro vs. in silico; Table S5: Nucleotide percent identity between Clade C L segments; Table S6; Nucleotide percent identity and amino acid similarity between Clade C S segments.
