# Supplementary material for: Novel Oliveros-like Clade C Mammarenaviruses from Rodents in Argentina, 1990–2020
Source: Viruses. 2024 Feb 22;16(3):340. doi: 10.3390/v16030340 (PMC10976098; doi:10.3390/v16030340)
Supplement: Supplementary file 1 [file viruses-16-00340-s001.zip › viruses-2820784-supplementary.pdf]

Supplemental Figures and Tables

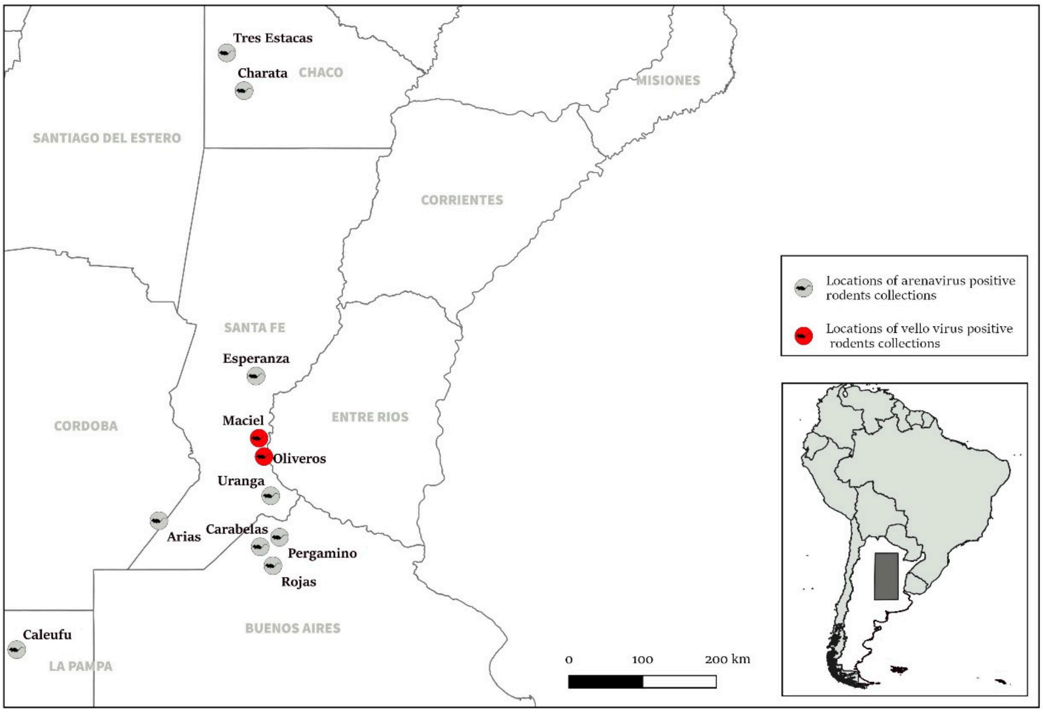

Figure S1. Distribution of rodent surveillance in Argentina between 1990 and 2019.

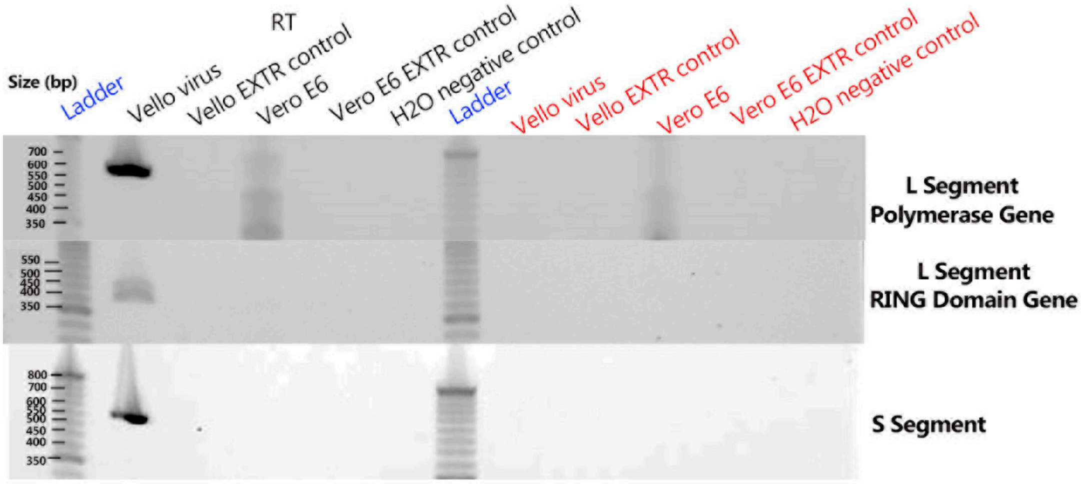

Figure S2. RT-PCR confirms the presence of the Vello virus genome. Amplicons were generated from the L segment - Polymerase gene (top), and RING domain gene (middle) and S segment (bottom). Amplicon sizes for the Polymerase gene, RING domain gene, and S segment were 642bp, 441bp, and 559bp

respectively. Reactions were repeated with an inactivated reverse transcriptase (indicated by "NO RT") to confirm that amplicons were from viral RNA and not a DNA template.

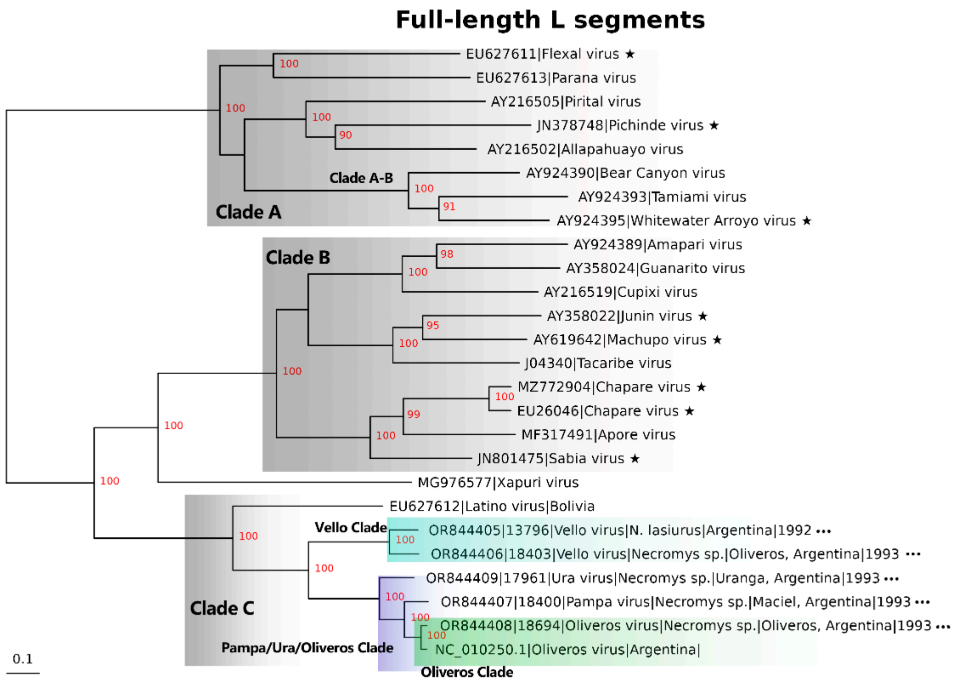

Figure S3: Inferred nucleotide-based phylogenetic relationships generated by maximum likelihood using full-length L segments belonging to all known New World viral genomes within the *Mammarenavirus* genus. Major clades are labeled, trees are midpoint rooted, and bootstrap support (n=1000 iterations) is highlighted in red on each node. Black stars indicate NW *Mammarenaviruses* known to cause human infections. Three dots indicate sequences generated in this work.

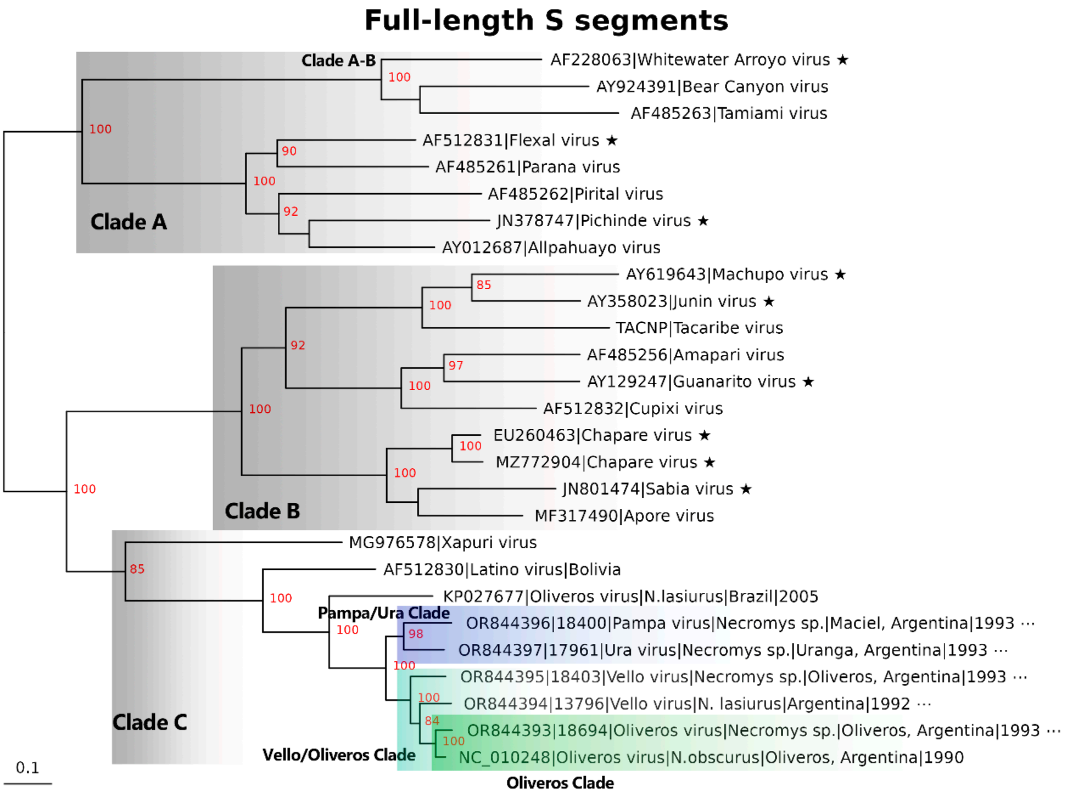

Figure S4: Inferred nucleotide-based phylogenetic relationships generated by maximum likelihood using full-length S segments belonging to all known New World viral genomes within the *Mammarenavirus* genus. Major clades are labeled, trees are midpoint rooted, and bootstrap support (n=1000 iterations) is highlighted in red on each node. Black stars indicate NW *Mammarenaviruses* known to cause human infections. Three dots indicate sequences generated in this work.

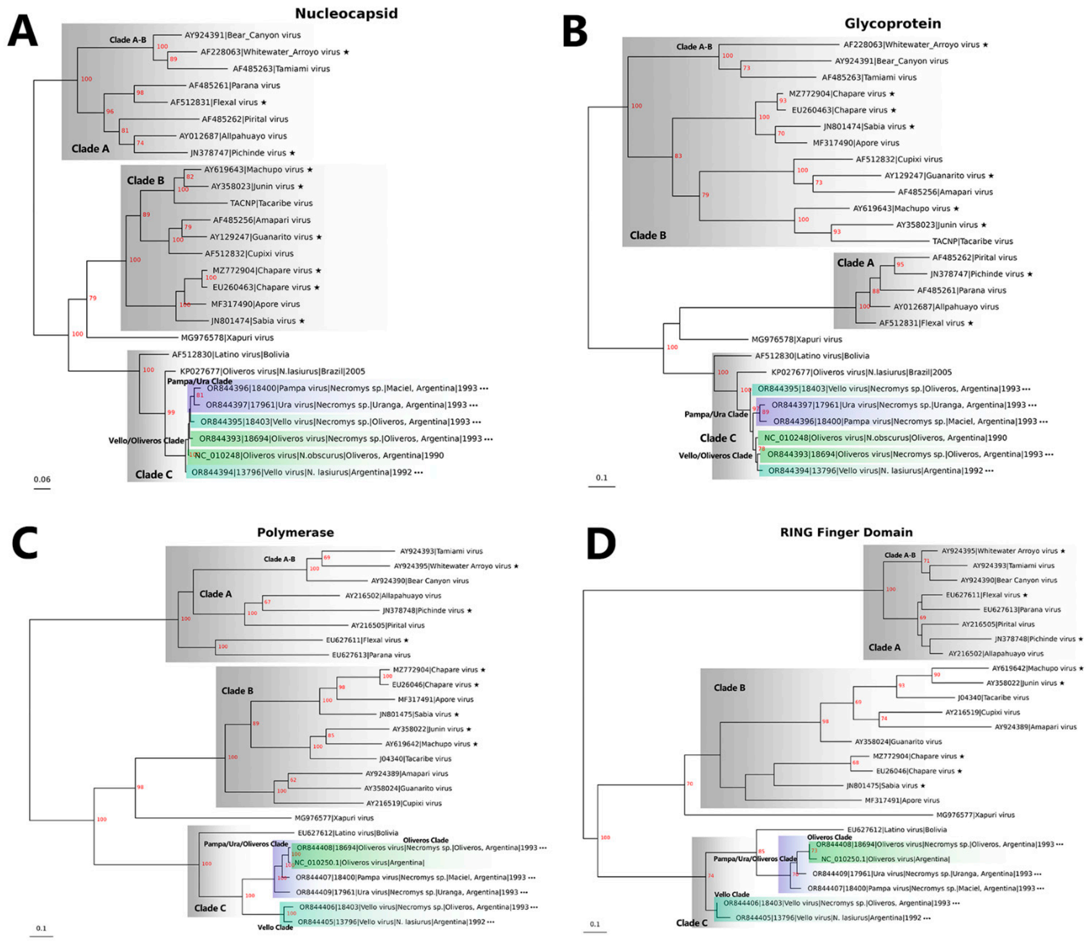

Figure S5: Inferred amino acid-based phylogenetic relationships generated by maximum likelihood using full-length S and L segments belonging to all known New World viral genomes within the *Mammarenavirus* genus. Trees based on the translations of the coding region for the nucleocapsid protein (panel A), glycoprotein (panel B), polymerase (panel C), and RING domain (panel D) are outlined. Major clades are labeled, trees are midpoint rooted, and bootstrap support (n=1000 iterations) is highlighted in red on each node. Black stars indicate NW *Mammarenaviruses* known to cause human infections. Three dots indicate sequences generated in this work.

Table S1. Comparison of L segment nucleotide percent identity between full-length Clade C *Mammarenavirus* sequences generated at Argentine National Institute of Human Viral Diseases (green) and Centers for Disease Control and Prevention (blue).

	Oliveros virus, 18694	Vello virus, 13796	Oliveros virus, 18403	Pampa virus, 18400	Ura virus, 17961	CDC
Oliveros virus, 18694	99.80%					
Vello virus, 13796		99.70%				
Oliveros virus, 18403			99.60%			
Pampa virus, 18400				99.30%		
Ura virus, 17961					100%	
INEVH	Full L-segment nucleotide percent identity					

Table S2. Comparison of S segment nucleotide percent identity between full-length Clade C Mammarenavirus sequences generated at Argentine National Institute of Human Viral Diseases (green) and Centers for Disease Control and Prevention (blue).

	Oliveros virus, 18694	Vello virus, 13796	Vello virus, 18403	Pampa virus, 18400	Ura virus, 17961	CDC
Oliveros virus, 18694	99.96%					
Vello virus, 13796		100%				
Vello virus, 18403			99.90%			
Pampa virus, 18400				-		
Ura virus, 17961					96.40%	
INEVH	Full S-segment nucleotide percent identity					

Table S3. Summary of *Mammarenavirus*-positive specimens

RNA ID	Species	Locality	Year Captured	Tissue type	Diagnosis methos	Diagnostic result	Next generation Result		Sanger S segment		Sequence number (F-R)
							S coverage	L coverage			
809227	Necromys lasiurus	Unknown	1992	Unknown	ELISA and NGS	Positive	100%	97.80%			
3335	Necromys sp.	Tres Estacas	2014	Lung	16V-19C (primers)	Positive	0%	0%	16.80%		4273-4274
1353	Akodon dolores	Caleufú	2013	Blood w/o heparin	16V-19C (primers)	Positive	0%	0%	16.70%		3795
1343	Necromys benefactus	Caleufú	2013	Blood w/o heparin	16V-19C (primers)	Positive	0%	0%	4.80%	16.80%	3790-3791
3414	Necromys sp.	Maciel	1993	10% Brain suspension	ELISA	Positive	99.80%	100%			
3400	Necromys sp.	Arias	2019	Brain	ELISA	Positive	0%	59.20%			
125	Necromys sp.	Uranga	1993	10% Brain suspension	16V-19C (primers)	Positive	100%	99.90%	16.1		802-803
36	Necromys sp.	Oliveros	1993	10% Brain suspension	16V-19C (primers)	Positive	99.90%	35.60%	16.8		790-791
92	Necromys sp.	Oliveros	1993	10% Brain suspension	16V-19C (primers)	Positive	99.9	100.00%			
91	Necromys benefactus	Rojas	1993	10% Brain suspension	16V-19C (primers)	Positive	0%	0%	14.7		972
90	Necromys benefactus	Pergamino	1993	10% Brain suspension	16V-19C (primers)	Positive	0%	0%	16%		948
88	Necromys benefactus	Pergamino	1992	10% Brain suspension	16V-19C (primers)	Positive	0%	0%	16.70%		946
1354	Akodon dolores	Caleufú	2013	Blood w/o heparin	16V-19C (primers)	Positive	0%	0%		6.3	3247
81	Necromys benefactus	Oliveros	1993	Blood 14 dpi	16V-19C (primers)	Positive	0%	0%			
1541	Akodon sp.	Charata	2013	Blood w/o heparin	142LAT-930LAT (pr	Positive	0%	0%			
3408	Necromys sp	Oliveros	1999	Brain	ELISA + JUNV	Positive	0%	0%			
3397	Necromys sp	Esperanza	2017	Brain	ELISA + JUNV	Positive	0%	0%			
3398	Necromys sp	Esperanza	2017	Brain	ELISA + JUNV	Positive	0%	0%			
3399	Necromys sp	Esperanza	2017	Brain	ELISA + JUNV	Positive	0%	0%			
3400	Necromys sp	Carabelas	2020	Brain	ELISA + JUNV	Positive	0%	0%			
3401	Necromys sp	Carabelas	2020	Brain	ELISA + JUNV	Positive	0%	0%			
3402	Necromys sp	Carabelas	2020	Brain	ELISA + JUNV	Positive	0%	0%			
3403	Necromys sp	Carabelas	2020	Brain	ELISA + JUNV	Positive	0%	0%			
3404	Necromys sp	Carabelas	2020	Brain	ELISA + JUNV	Positive	0%	0%			
3405	Necromys sp	Carabelas	2020	Brain	ELISA + JUNV	Positive	0%	0%			
3406	Necromys sp	Carabelas	2020	Brain	ELISA + JUNV	Positive	0%	0%			

Table S4. Comparison of Vello virus *in vitro* sequence compared to Vello virus *in silico* sequence.

Amplicon	*Nucleotide Percent Identity*	*Amino acid Percent Identity*	*Amino acid Percent Similarity*
S-segment	100%	100%	100%
Polymerase gene	99.6%	100%	100%

Table S5. Comparison of L segment nucleotide percent identity between full-length and partial Clade C *Mammarenavirus* sequences. Red highlighting indicates a percent similarity below the ICTV threshold for *Mammarenavirus* species demarcation.

	Oliveros virus, NC_010250.1	Oliveros virus|18694, OR844408	Pampa virus|18400, OR844407	Ura virus|17961, OR844409	Vello virus|18403, OR844406	Vello virus|13796, OR844405	Latino virus, EU627612
Oliveros virus, NC_010250.1							
Oliveros virus|18694, OR844408	96.6%						
Pampa virus|18400, OR844407	89.4%	89.2%					
Ura virus|17961, OR844409	83.0%	82.8%	83.1%				
Vello virus|18403, OR844406	**68.8%**	**68.6%**	**70.8%**	**69.6%**			
Vello virus|13796, OR844405	**70.4%**	**69.8%**	**70.8%**	**70.4%**	82.9%		
Latino virus, EU627612	67.4%	64.0%	64.0%	64.2%	64.2%	64.2%	
	**Full L-segment nucleotide percent identity**						

Table S6. Comparison of S segment nucleotide percent identity (bottom) and NP amino acid percent identity (top) between full-length Clade C *Mammarenavirus* sequences. Red highlighting indicates a percent similarity below the ICTV threshold for *Mammarenavirus* species demarcation.

	Oliveros virus, NC_010248	Oliveros virus|18694, OR844393	Vello virus|13796, OR844394	Vello virus|18403, OR844395	Pampa virus|18400, OR844396	Ura virus|17961, OR844397	Latino virus, AF512830	
Oliveros virus, NC_010248		98.4%	98.7%	97.9%	96.2%	96.2%	80.7%	NP amino acid sequence identity
Oliveros virus|18694, OR844393	95.0%		97.5%	97.3%	95.2%	95.7%	81.0%	
Vello virus|13796, OR844394	90.10%	87.9%		97.5%	95.5%	96.1%	80.9%	
Vello virus|18403, OR844395	88.50%	87.9%	87.5%		95.3%	95.9%	81.2%	
Pampa virus|18400, OR844396	84%	83.2%	83.0%	82.6%		96.1%	80.9%	
Ura virus|17961, OR844397	**73.70%**	83.9%	**73.7%**	**72.7%**	**75.2%**		80.7%	
Latino virus, AF512830	70.70%	69.30%	70.60%	70.10%	70.30%	62.10%		
	Full S-segment nucleotide identity							
